# The social context of smoking: A qualitative study comparing smokers of high versus low socioeconomic position

**DOI:** 10.1186/1471-2458-10-211

**Published:** 2010-04-27

**Authors:** Christine L Paul, Samantha Ross, Jamie Bryant, Wesley Hill, Billie Bonevski, Nichola Keevy

**Affiliations:** 1 Centre for Health Research and Psycho-oncology (CHeRP), Cancer Council New South Wales, University of Newcastle, and Hunter Medical Research Institute, Callaghan, 2308, Australia; 2 Urbis, Sydney, 2000, Australia

## Abstract

**Background:**

The reductions in smoking prevalence in a number of industrialised countries are accompanied by a strong social gap and associated health inequality. Groups such as the World Health Organisation emphasise the importance of exploring potential causal factors for smoking such as socio-economic context & position. There has been little effort to compare the social context of smoking for smokers of high versus lower socio-economic position (SEP) to consider how tobacco control efforts might reduce smoking-related health inequality.

**Method:**

Purposive sampling was used to recruit participants for eight focus groups. The groups were segregated by age, gender and SEP. Samples were selected from suburbs within the Sydney metropolitan area defined as either high or low SEP based on the Socio Economic Index for Areas. Emergent themes were analysed according to Poland's six dimensions of the social context of smoking. Differences according to SEP, age group and gender were explored.

**Results:**

While there was commonality in social experiences for smokers across groups, some important aspects of the social context of smoking varied. Smokers of high SEP appeared to be aware of particular social pressures not to smoke on five of the six social context dimensions (power, body, identity, consumption and place). Not only were some of those pressures absent for low SEP participants, there were additional influences within the social context which were pro-smoking.

**Conclusions:**

In order to narrow the health inequality gap associated with smoking, it is important to take account of the more pro-smoking social context experienced by low SEP smokers. Suggestions are made regarding social marketing campaigns, support for quit assistance and approaches to the regulation of smoking which may assist in minimising smoking-related health inequality.

## Background

### Tobacco-related burden of disease

Tobacco use is the single largest preventable cause of disease and premature death [1]. Public health campaigns have been associated with a reduction in the prevalence of tobacco use, particularly in a number of developed countries [1]. Australia for example, has seen the prevalence of tobacco smoking drop from 27.1% of men and 23.2% of women in 1985 [2,3] to 16.6% overall in 2007 [3].

### Relationship between smoking and socio-economic position (SEP)

Despite reductions in smoking rates, a strong socioeconomic gradient for smoking prevalence exists in a number of countries and has persisted over time [4,5]. In Australia, rates of smoking in groups of lowest socio-economic status (SEP) are 26% compared to 13% for those in the highest SEP category [3]. While socially disadvantaged smokers attempt to quit at rates similar to those of all smokers, they are less likely to be successful in their quit attempts [6,7]. A number of factors appear likely to contribute to poor success rates for these populations including higher rates of nicotine dependence [8], fewer prompts to quit [9], using smoking as a particular means of coping with daily stress and anxiety [7,10] and social or environmental factors [11].

### The importance of understanding the social context of smoking

The causal pathways framework used by the WHO Commission on the Social Determinants of Health emphasises the importance of looking 'up-stream' at causal factors associated with smoking such as socio-economic context and differential exposure to smoking cues via the social & physical environment [12]. It is possible that low SEP groups experience tobacco marketing and tobacco control efforts differently than their higher SEP counterparts. It may be that tobacco control campaigns could be modified to address any such differences and so have a greater impact with low SEP groups.

Foundational theories such as Bandura's social cognitive theory [13] emphasized the influence of social and environmental factors over individuals' judgement of and regulation of their own behaviour. This is reflected in the emphasis on the concept of 'de-normalisation' which underpins various aspects of tobacco control work such as advocacy for restrictions on smoking in public [14].

### Theoretical perspectives on the social context of smoking

A range of factors relating to social and cultural contexts have been proposed to facilitate smoking behavior [15,16]. The transdisciplinary framework of Unger et al (2003) [16] emphasises the importance of the socio-cultural context via interactions between social norms and culture.

Poland et al (2006) [15] identify six dimensions of the social context which can assist in understanding how socio-spatial disparities may influence smoking behaviour or hamper tobacco control efforts. These dimensions are: i) power: - how social and geographic patterns of smoking parallel the effects of marginalisation and disadvantage; ii) the body - physicality and sociality associated with smoking; iii) collective patterns of consumption - smoking occurring within a social group in terms of what is economically and socially feasible; iv) social identity - establishing and expressing difference among and between social groups; v) desire or pleasure associated with the act of smoking; and vi) smoking as a social activity embedded in place.

Previous qualitative explorations of SEP and the social context of smoking generally have included only low SEP smokers and have failed to concurrently examine other important potentially intercepting factors such as age and gender [11,17,18]. While there has been some description of the social context of smoking for smokers of low SEP [11,17,19], there has been no qualitative exploration of how SEP interacts with the social context of smoking.

This study aimed to qualitatively examine whether smokers' experiences of the social context of smoking differed according to their age, gender and socio-economic position, using Poland's framework as a guide.

## Method

### Sample

Purposive sampling was used to obtain samples of high and low SEP people for specific age groups (18-40 years, more than 40 years) and gender. Purposive sampling is designed to reflect the diversity within a population rather than statistical generalisability or representativeness [20]. In this case, purposive sampling involved seeking smokers who lived in suburbs of relevant SEP (and would therefore have some commonality of place as per Poland's framework) and were of an employment or income status reflective of that SEP.

Suburbs within the Sydney metropolitan area of NSW, Australia were classified as high or low SEP based on the Australian Bureau of Statistic's Socio Economic Index for Areas [21]. The SEIFA consists of four separate indexes which measure different aspects of the social and economic conditions in an area, providing a more holistic measure of socio-economic status than would be given by, income or unemployment alone. High SEP areas according to SEIFA would generally have residents on relatively high incomes, with high educational attainment and full employment compared to low SEP areas. Four of the highest SEP and four of the lowest SEP suburbs were identified and residents of those areas were sought. Each participant was also required to be a self-reported current smoker (daily or occasional smoker) who had made a quit attempt of any duration within the last 12 months. An informal confirmation of individual SEP in terms of employment or income status was later gained during the course of the focus group discussions. An informal process was used to verify personal eligibility given the importance of gaining spontaneous discussion about SEP-related issues. Where it was apparent that the SEP group composition varied from that desired, a replacement group was sought.

### Procedure

Recruitment was conducted through a professional recruiter to facilitate direct access to relevant participants. The recruitment agency contacted individuals using existing lists and networking techniques to obtain a sample of individuals who met the eligibility criteria described above (resident in the selected suburb, a current smoker who had made a quit attempt in previous 12 months and in the required age group). The study was described as "a study with smokers to talk about the things around them that encourage smoking" Participants were not aware that the groups were segregated according to SEP.

Eight focus groups lasting one and a half hours were conducted over a three week period in May 2008. The venues were local community facilities. The facilitators (SR, WH) were trained and highly experienced in conducting health-related and market research. At the conclusion of each group, all participants received $90 reimbursement to cover attendance costs. The study received approval from the Human Research Ethics Committee of the University of Newcastle.

### Discussion Guide

The focus groups followed a discussion guide which addressed smoking behaviour and history (the general circumstances in which smoking occurred). Current and future smoking environments including the physical, social and regulatory context were raised to facilitate consideration of each dimension posited by Poland et al (2006) [15]. Hypothetical scenarios such as government bans on cigarette sales were used to prompt discussions about the regulatory context. Environmental factors discussed included locations where smoking occurred with greater or lesser frequency and intensity, the degree of social and physical amenity for smoking in those environments, types of individuals who smoked near or with the participants; and how each of these impacted on desire and ability to quit. Additional issues were discussed but are not presented.

### Analysis

Audio-tapes of interviews were transcribed verbatim and checked for errors. Transcripts were studied and information pertaining to perceptions of social and environmental aspects of smoking behaviour was extracted. These were categorised by age, gender and SEP. Thematic analysis to identify emergent themes relating to the study aims was conducted by the focus group facilitator (SR). A second author (CP) coded the data to verify thematic analysis, extract any additional items of data and compare emergent themes with the six dimensions in Poland's framework.

Only the themes relating to the stated aims are presented here. Focus was given to describing differences by SEP, age or gender rather than themes common to all groups, as there is sufficient qualitative data on smokers' experiences in general or within low SEP groups. Commonalities are therefore, only reported very briefly at the beginning of the results to provide context for the main results.

## Results

### Sample

Eight focus groups (two within each age group, gender and SEP) were conducted with four to eight participants in each group. One focus group facilitator made a subjective assessment of whether the recruited participants were appropriate to the expected SEP for that suburb. One group which did not appear to contain appropriate participants was discarded and a replacement group was recruited. Although all participants had made some attempt to quit in the past 12 months, most participants did not report immediate plans to quit smoking.

### Common themes which did not differ by SEP, age or gender

Themes which were common across SEP groups, age groups and gender included: a sense of relaxation, enjoyment and satisfaction when smoking (body & pleasure); home as a place where smoking is acceptable and can occur in privacy and comfort (consumption); sometimes feeling isolated by their smoking behaviour (identity) and a sense of 'camaraderie in exile' among smokers in designated smoking areas (consumption). Certain environments were considered 'smoke friendly' and so resulted in heavy smoking (place). Negative looks, judgemental attitudes and being "looked down on" were generally reported when smoking in public (power & identity). Seeing others smoking in public was considered permission to smoke (consumption).

*"When you see someone else it's like cool, I'll have one too" *[Low SEP older female]

*"Finally someone said it and we all go out to smoke" *[High SEP older male]

### Differences by age or gender

Some experiences or perceptions appeared to be related to age or gender and not SEP, as described below.

#### Power

##### Younger smokers are more accepting of smoking restrictions

The impact of legislation to restrict smoking was generally accepted by younger groups as an expected part of their lives. Older groups were less accepting, with some expressing outrage at the removal of their 'rights' and a tendency to respond angrily to public comments about their smoking.

#### Body

##### Smoking associated with nostalgic images of sophisticationfor older groups

Men and women in the older age groups reported a nostalgic sense that smoking was once *"cool" *&*"sophisticated*", even though that was no longer considered to be the case.

##### Weight control is a motivator for women's smoking

Women in all groups nominated weight control as in important reason for continuing to smoke, while no males nominated weight as an issue.

#### Collective Patterns of Consumption

##### Peer influences strong for men

Older men noted that they smoked most frequently when alone, but more heavily when with other smokers. Young men in both SEPs and older high SEP males suggested that social situations with their 'mates' prompted them to smoke more, or smoke when they had not intended to. Males reported a strong influence on their behaviour from their smoking friendship groups regardless of age, whereas this was not noted in the female groups.

##### Smoking is a more social activity for women than for men

Women reported that they tended to smoke most frequently when in the company of other smokers. Men reported that they smoked alone on a regular basis, but more heavily when in company. Older men reported that rather than the social activity smoking had been when they were younger, it had come to be a solo behaviour due to the need to smoke. Older men of both higher and lower SEP reported that social groupings often predominantly involved non-smokers, leading to discomfort in social situations if they needed to smoke.

#### Identity

No age or gender related differences were observed in comments relating to identity

#### Pleasure

##### Enjoyment of smoking lessens with age

Men and women in the younger age groups nominated their enjoyment of smoking and its association with leisure as strong factors in their continued smoking. Participants in the younger age groups discussed the act of smoking as being enjoyable in itself and reported its association with 'having a good time'.

"*I'm not addicted, I enjoy it. But I can't quit - I enjoy the taste*" [High SEP young female]

For the older groups, the enjoyment factor was less prominent. Older women noted the effects of both enjoyment and addiction, while older men focussed on habit and addiction as drivers for their behaviour. Male participants in the older-age groups reported that they enjoyed smoking less than they used to due to the increased restrictions on smoking during their lifetime.

#### Place

##### Changes in smoking restrictions over time have reduced consumption for older groups

Both men and women in the older age groups reported smoking less often than they had earlier in their lives due to the increased environmental restrictions on smoking. Older low SEP men reported smoking and gambling remained a comfortable pairing given the emergence of outdoor gaming areas.

##### Home is a guilt-free place to smoke for young women

Young women in particular appeared to identify home as the place where they felt less guilty or less judged for their smoking behaviour.

"*I find myself waiting to get home" *[High SEP young female]

*"You don't feel like you're doing the wrong thing" *[Low SEP young female]

### Differences by Socio-Economic Position

Differences were observed between higher and lower SEP groups in some social and environmental experiences in relation to work, home and social environments; and perceptions about the prevalence of smoking.

#### Power

##### Government restrictions empower quitting for high SEP groups but may be circumvented by low SEP groups

High SEP groups generally reported that increased regulation such as bans on cigarette sales could be potentially beneficial in supporting their quitting attempts, while low SEP groups tended to report that rather than quitting they would need to seek alternative sources of cigarettes such as *"the black market" *or alternative sources of enjoyment ("*something to fill the void*" - Low SEP Young Male). Low SEP groups, particularly the young, had previously been exposed to free sampling of NRT and may expect subsidy for these products:

"*The government would have to help" *[Low SEP Older Female].

High SEP groups did not report a need for financial support in relation to quitting.

#### Body

##### Act of smoking incongruous with walking in public for high SEP groups

Higher SEP groups described a sense that it felt physically incongruous to smoke while walking in public.

*"Walking down the street I sometimes find myself just needing to have one in transit and I feel really uncomfortable." *[High SEP Young Females]

*"It looks a bit odd." *[High SEP Young Females]

*"In fact I think it looks really bad walking along the street smoking" *[High SEP Older Males]

This view which was not expressed by low SEP groups.

#### Collective Patterns of Consumption

##### Smoking prevalence perceived to be higher by low SEPgroups

While high SEP participants reported a perception of decreased smoking prevalence in their social circle and the community over time, lower SEP men and women had not noticed any such decrease.

*"The majority of people smoke. I don't' know if it's half/half but most people I know do smoke." *[Low SEP, Older Female]

##### Smoking is a more frequent social activity for younger low SEP groups

Young low SEP women reported that their social groupings were often made up predominantly of smokers, making their social environment very conducive to smoking. Young low SEP women enjoyed getting together and smoking with their group of friends at home. Young high SEP women reported being highly conscious of social taboos about smoking. Smoking at friends' houses was either very comfortable or very uncomfortable, depending on the smoking status of the host and guests. Older high SEP males generally found social gatherings resulted in being surrounded by non-smokers, and did not smoke when socialising in people's homes:

*"Other people's homes, definitely not. Never" *[High SEP Older Males]

##### Perceived acceptability of smoking in observable groups is greater for low SEP groups

High SEP groups emphasised a sense of alienation and being ostracised when having to leave a group to smoke. High SEP older women also reported that in their view groups of smokers congregating outdoors looked *"horrible"*. Low SEP groups, while also reporting social isolation due to the need to smoke outside, reported it was accepted to smoke in groups such as with mothers at the school gate (Low SEP Young Females), as a part of socialising or as a way to make new acquaintances (Low SEP Young Males).

#### Identity

##### Smoking seen as foolish and shameful by high SEP groups

Young high SEP women reported a sense of shame and embarrassment. Purchasing cigarettes was considered to indicate foolishness, poor self-control, and an unhealthy lifestyle. Older high SEP males also interpreted smoking as being indicative of foolishness.

"It's just so bad for you and you feel like such an idiot."

*"It's almost like we don't care about ourselves." *[High SEP Young females]

*"When I see people out and about smoking I look at it and think they're idiots" *[High SEP older male]

High SEP young males also reported that now they were adults, smoking was no longer "cool". Both low and high SEP young men reported smelling of smoke created a bad impression at work, while older high SEP males reported a sense of shame when smelling of cigarettes when with a non-smoking woman.

##### Minimising observable indicators of smoking for high SEP women

Older high SEP women emphasised the need to be a 'clean' smoker - someone who did not smoke inside or near others, did not have ashtrays and did not smoke in the car. The need for smoking to be a hidden activity which did not impact on other aspects of their lives was considered important for this group.

#### Place

##### Low SEP work environments more conducive to smoking

In each of the low SEP groups, there were reports of the work environment being conducive to smoking. These participants tended to have workplaces that allowed them to take regular breaks for smoking. Participants from the lower SEP female groups (mainly office and retail environments) reported an absence of anti-smoking pressures at work and reported taking regular smoking breaks with groups of co-workers throughout the day, sometimes as an alternative to meals.

Low SEP male groups, particularly older males, included some participants who worked outdoors where smoking was unrestricted. These participants reported that lack of restriction resulted in frequent smoking at work.

*" As soon as I walk into work, clock on and there is a table out the back, just like this for all the smokers, and you can catch up on all the gossip and the rest of it." *[Low SEP Older Male]

For younger low SEP men there were mixed reports of environments either conducive or non-conducive to smoking, depending on their type of workplace. Younger male participants in both high and low SEP groups who were office workers reported being highly conscious of the negative impacts of their smoking behaviour at work. In those environments smoking was met with negative responses by co-workers and management:

"*Work colleagues are friendly until they see you smoke, then things change" *[Low SEP young male]

"*Recently an email went round complaining about people... coming back having their breath and clothes smell like smoke- it's bad for clients" *[High SEP young male]

Similar pressures were reported by high SEP young females, including comments from colleagues encouraging them to quit and being offered monetary incentives by employers for quitting.

##### Low SEP suburban environments more conducive to smoking

The degree of acceptability of smoking in open-air public places was also considered to vary by suburb or in relation to neighbourhood acceptance of smoking:

*"Over at (low SEP suburb) they couldn't give a bugger if you walk down the street smoking but in (high SEP suburb) it is a social stigma." *[High SEP older male]

*"When I know that the neighbours have all gone out ... I can sit in the backyard on a chair with a nice book, a bottle of red and a packet of fags*..." [High SEP older male]

## Discussion

The focus group data presented here suggest that while there was considerable commonality in the social context of smoking for all smokers, some important aspects of the social context varied according to age, gender and socio-economic position. Among lower SEP participants a greater variety of pro-smoking factors were found to be present, and conversely, among higher SEP participants a greater variety of anti-smoking factors were apparent.

### Age and Gender

Age appears to be a major determinant influencing the social context of smoking. Older participants expressed a sense of change over time which had shaped their perspectives. Older smokers reported the recollection of positive bodily associations with smoking, reduced pleasure associated with smoking and expressed an element of anger towards increased smoking restrictions over time. Quantitative research suggests older smokers are also more likely to hold self-exempting beliefs about health effects of smoking [22]. Therefore, tobacco control efforts which address the effects of past context on current smoking-related beliefs and behaviour may assist older smokers to quit.

Gender-related differences suggested that women, particularly young women may find smoking to be a social bond or at least a more positive social experience than is the case for men. For women, their perceptions about the enjoyment of smoking seemed to persist with age, but not so for men. This may in part explain the rise in smoking among women in previous decades, even once male smoking rates had begun to decline [23]. Tobacco control efforts which further reduce the potential for social contact while smoking would therefore be beneficial. Further efforts to reduce smoking in or near the home appear worthwhile on this basis.

### Socio-economic Position & Tobacco Control Initiatives

A consideration of the emergent themes in the context of Poland's six dimensions of the social context of smoking illustrates the disparity between high and low SEP smokers in terms of the context in which their smoking behaviour occurred. Smokers of high SEP appeared to be conscious of particular social pressures not to smoke on five of the six dimensions. Not only were some of those pressures absent for low SEP participants, there were additional influences which were relatively pro-smoking in terms of the dimensions of power, consumption and place. In terms of Unger's (2003) [16] framework of the cultural context of smoking, the social norms around tobacco use appear to be more pro-smoking for low SEP smokers than they are for high SEP smokers at the personal, community and societal levels. It is likely that population-wide tobacco control efforts have more opportunity for synergistic effects among high SEP groups. The potentially more conducive social context of smoking for low SEP participants may help to explain why low SEP smokers want to quit but are less likely to succeed [6,7].

Differences in perceived *power *may act to mediate restrictions on smoking in that some low SEP smokers may feel more empowered to seek alternatives, more in need of substances such as tobacco, and felt themselves to be without the economic resources they perceived were required to quit. It may be that increases in the availability of effective quit assistance to those of low SEP may be beneficial. Alternatively, additional promotion regarding the high proportion of smokers who quit without assistance may be helpful to counter the addiction-focussed messages contained in the marketing of nicotine replacement therapies.

The SEP differences in *collective patterns of consumption *suggest a need to increase the reach of smoking restrictions into the locations where socialising occurs for low SEP groups. Social marketing efforts could be directed towards decreasing the social acceptability of smoking in a greater range of social contexts, particularly those relevant to low SEP groups. Increasing awareness of the declining prevalence of smoking, particularly for low SEP groups may also be beneficial. Given the negative *identity *associated with smoking by high SEP groups, it may be time to consider ways to create negative social connotations for smoking which have relevance for low SEP groups.

The difference between high and low SEP experiences of *place *in terms of work and urban environments suggests a need to consider how smoking might be regulated in a greater range of work environments, such as outdoor work environments. Where smoking-related local ordinances may differ by SEP (e.g. a poor suburb has fewer restrictions on outdoor smoking), efforts of tobacco control advocates might be directed toward these areas, rather than working more closely with 'friendly' areas.

### Study Limitations

The qualitative nature of the study and the associated purposive sampling process cannot provide representative data on the experiences of people living in high versus low SEP environments. Clearly quantitative approaches are required to identify whether the apparent differences found here are the case for smoking more generally. Observing the number or variety of emergent themes which are pro-smoking or anti-smoking is not the same as quantifying their effects on participants and may represent an oversimplification of the issue.

## Conclusions

In order to close the health inequality gap associated with smoking, it is important to take account of the more pro-smoking social environment experienced by low SEP smokers. Social marketing campaigns, support for quit assistance and approaches to the regulation of smoking should consider the social context of smoking for low SEP groups in order to minimise smoking-related health inequality. Such developments will need to be cognisant of other potential drivers for quitting identified in the research, some of which were independent of SEP. Future quantitative research to confirm these qualitative findings would be useful.

## Competing interests

The authors declare that they have no competing interests.

## Authors' contributions

CP designed the study, co-analysed the data, and drafted the manuscript. SR conducted focus groups, analysed data and was involved in manuscript preparation. JB was involved in study design, data collection and manuscript preparation. WH conducted focus groups and was involved in manuscript preparation. BB was involved in concept development and manuscript preparation. NK was involved in data collection and manuscript preparation. All authors read and approved the final manuscript.

## Pre-publication history

The pre-publication history for this paper can be accessed here:

http://www.biomedcentral.com/1471-2458/10/211/prepub
